# Involvement of microRNAs-449/FASN axis in response to trastuzumab therapy in HER2-positive breast cancer

**DOI:** 10.1186/s10020-025-01163-z

**Published:** 2025-03-25

**Authors:** Ana Lameirinhas, Sandra Torres-Ruiz, Iris Garrido-Cano, Cristina Hernando, María Teresa Martínez, Ana Rovira, Joan Albanell, Sandra Zazo, Federico Rojo, Begoña Bermejo, Ana Lluch, Juan Miguel Cejalvo, Eduardo Tormo, Pilar Eroles

**Affiliations:** 1 INCLIVA Biomedical Research Institute, Valencia, 46010 Spain; 2 Instituto Interuniversitario de Investigación de Reconocimiento Molecular y Desarrollo Tecnológico (IDM), Universidad Politécnica de València, Universidad de Valencia, Valencia, 46022 Spain; 3 Department of Medical Oncology, Hospital Clínico Universitario de València, Valencia, 46010 Spain; 4 Center for Biomedical Network Research on Cancer (CIBERONC), Madrid, 28019 Spain; 5 Department of Medical Oncology, Hospital del Mar, Barcelona, 08003 Spain; 6 Cancer Research Program, IMIM (Hospital del Mar Medical Research Institute), Barcelona, 08003 Spain; 7 Department of Pathology, Fundación Jiménez Díaz, Madrid, 28040 Spain; 8 Department of Medicine, Universidad de Valencia, Valencia, 46010 Spain; 9 Department of Physiology, Universidad de Valencia, Valencia, 46010 Spain

**Keywords:** Breast cancer, HER2, microRNAs-449, FASN, Trastuzumab

## Abstract

The anti-HER2 monoclonal antibody trastuzumab and new derivative formulations are the standard treatment for HER2-positive breast cancer. However, after 1 to 5 years of treatment, some patients acquire resistance to therapy, leading to relapse. The microRNA-449 family members were downregulated in HER2-positive breast cancer cell lines and low levels were associated with patients’ worse prognosis. Moreover, trastuzumab-resistant HER2-positive breast cancer cell lines showed lower microRNAs-449 and higher Fatty Acid Synthase (FASN) expression, compared to sensitive cell lines. The direct regulation of *FASN* by microRNA-449a and microRNA-449b-5p was demonstrated. Moreover, microRNAs-449 overexpression and *FASN* inhibition decreased cell proliferation and sensitized cells to trastuzumab treatment by inhibiting the PI3K/AKT signaling pathway. Together, these results suggest the microRNAs-449/FASN axis as a potential therapeutic target in combination with anti-HER2 agents to overcome trastuzumab resistance and to improve treatment response in HER2-positive breast cancer patients.

**Supplementary Information:**

The online version contains supplementary material available at 10.1186/s10020-025-01163-z.

## Introduction

Breast cancer (BC) is the most common malignancy among women worldwide and it remains a public health concern (Katsura et al. 2022). Approximately, 15 to 20% of BCs show Human Epidermal Growth Factor Receptor 2 (HER2) overexpression. HER2 amplification leads to the overactivation of proto-oncogenic signaling pathways, such as Phosphatidyl Inositol 3 Kinase (PI3K) and Mitogen-activated Protein Kinase (MAPK) pathways, that regulate cell proliferation, invasion, migration, angiogenesis, and apoptosis, leading to uncontrolled cancer cell growth (Yarden and Sliwkowski 2001). Nowadays, the standard therapy for HER2-positive (HER2+) BC patients is a combination of anti-HER2 targeted therapy agents such as the monoclonal antibody trastuzumab (TZ), and chemotherapy (Valachis et al. 2011). TZ is a monoclonal antibody that binds to the extracellular domain of HER2, blocking activation of downstream signaling (Saha and Lukong 2022). Nevertheless, despite the good initial response of this combined therapy, after 1–5 years of treatment a significant percentage of HER2 + patients relapse and/or develop metastases due to acquired resistance (Chumsri et al. 2019).

MicroRNAs (miRNAs) are a class of small noncoding RNAs (19–25 nucleotides in length), which are involved in the posttranscriptional regulation of gene expression at the messenger RNA (mRNA) level (Bartel 2004). MiRNAs play an important role in many biological functions, such as development, cell differentiation, embryogenesis, and metabolism (Huang et al. 2011). Furthermore, miRNAs dysregulation plays a critical role in different pathologies, including cancer (Paul et al. 2018).

The miRNA-449 family belongs to the miRNA-34/miRNA-449 superfamily of miRNAs, located in the second intron of the Cell division cycle 20B (CDC20B) gene on chromosome 5q11.2, a susceptibility locus in cancer (Naydenov et al. 2022). This family is composed of miRNA-449a, miRNA-449b-5p, and miRNA-449c-5p and was found to be downregulated in several cancer types, including BC (Marcet et al. 2011; Yong-Ming et al. 2017). Therefore, miRNAs-449 role as tumor suppressors is suggested by their ability to target genes involved in cell proliferation, migration, invasion, apoptosis, and cell cycle (Bao et al. 2012). Furthermore, their role as biomarkers and therapeutic targets for various types of cancer and diseases has been proposed (Barati et al. 2024; Hosseinpour et al. 2023; Re et al. 2022; Torres-Ruiz et al. 2024).

Besides, miRNAs are also implicated in BC treatment resistance and response (Garrido-Cano et al. 2022). Several studies confirmed the miRNA-449 family’s implication in drug resistance, but there is no information about its role in the TZ response. A recent study demonstrated the role of miRNA-449a in sensitizing triple-negative BC cancer cells to tamoxifen through *ADAM Metallopeptidase Domain 22* (*ADAM22*) downregulation, and to olaparib through *survivin/Breast Cancer Gene 2* (*BRCA2*) suppression via *E2F Transcription Factor 3* (*E2F3*) knockout (Li et al. 2018; Vajen et al. 2022). Furthermore, another study suggested a role for the miRNA-449 family in doxorubicin sensitization by cell cycle-dependent cyclins modulation (Tormo et al. 2019). However, little is known about the role of miRNA-449a, miRNA-449b-5p, and miRNA-449c-5p in TZ response. Therefore, this study aims to evaluate the role of the miRNA-449 family in the modulation of TZ response in HER2 + BC.

## Materials and methods

### Cell lines

The MCF10A (RRID: CVCL_0598), BT474 (RRID: CVCL_0179), SKBR3 (RRID: CVCL_0033), and HEK293T (RRID: CVCL_0063) cell lines were obtained from American Type Culture Collection (ATCC, Manassas, USA). We used the lines until passage 30 and before the experiments, the mycoplasma test was performed with the MycoStrip kit (rep-mysnc-50, InvivoGen, France). The acquired TZ-resistant cell lines (BT474R and SKBR3R) were provided by Dr. Federico Rojo (Fundación Jiménez Díaz, Madrid) (Zazo et al. 2016). To obtain the resistant cells, the TZ-sensitive parental cell lines (BT474 and SKBR3) were exposed to increasing concentrations of TZ until reaching the resistance criterion, which was the maintenance of at least 80% of the cell viability after the TZ treatment.

Cells were grown in Dulbecco’s Modified Eagle Medium F12 (DMEM-F12) (Biowest, Nuaillé, France) medium supplemented with 10% Fetal Bovine Serum (FBS; Gibco, Carlsbad, USA), 1% penicillin-streptomycin (Biowest) and 1% L-glutamine (Biowest), at 37 °C, 5% CO_2_ in humid atmosphere conditions. In addition, BT474R and SKBR3R cell lines were maintained in complete medium containing 15 µg/mL TZ (Chemocare, Cleveland, USA), and the medium was renewed every 2–3 days.

### Gain and loss-of-function assays

Cells were cultured until they reached 80–90% confluence and transfected using OPTIMEM (Gibco) culture medium with Lipofectamine™ 2000 (Invitrogen, Carlsbad, USA), following the manufacturer’s instructions. For miRNAs-449 gain-of-function, the cell lines were transfected with 50 nM of miRNA-449a (#MC11127, Ambion, Austin, USA), miRNA-449b-5p (#MC11521, Ambion), and miRNA-449c-5p (#MC15616, Ambion) mimics. For *Fatty Acid Synthase* (*FASN*) loss-of-function, 100 nM of *FASN* small interfering RNA (si-RNA) (si-FASN, #107315, Ambion) was used to transfect the cells. A miRNA molecule (#4464059, Ambion) or siRNA molecule with no significant sequence similarity to mouse, rat, or human genetic sequences (#4390844, Ambion) were used as negative transfection controls, respectively. Transfection was validated by real-time quantitative PCR (RT-qPCR) and Western blot after 48 h and 72 h, respectively (**Supplementary Fig. 1**).

### WST-1 assay

Cell viability and proliferation were determined by WST-1 assay. For the cell proliferation assay, 10,000 cells/well were seeded in a 96-well plate and proliferation was determined at 24, 48, 72, and 96 h.

For cell viability assay, 2,500 cells/well were seeded in a 96-well plate and treated with TZ (15 µg/mL). Untreated cells were included as a control. Viability was determined after 7 days.

At the final endpoints, the cells were incubated for 2 h with colorless DMEM-F12 culture media (Biowest) and 7% WST-1 reagent (#K304-2500, Deltaclon, Madrid, Spain), the absorbance was measured at 450 nm and 650 nm in the plate reader SpectraMax Plus 384 (Thermo Fisher Scientific, Waltham, USA).

### RT-qPCR

Total RNA was extracted using TRIzol™ (Invitrogen) reagent, following the manufacturer’s instructions. NanoDrop Lite spectrophotometer (NanoDrop Technologies, Wilmington, DE, USA) was used for demining the RNA concentrations and purity ratios. 1000 ng of RNA were reversed transcribed using the High-Capacity cDNA Reverse Transcription kit (#4368813, Thermo Fisher Scientific) or TaqMan MicroRNA Reverse Transcription kit (#4366597, Thermo Fisher Scientific), following the manufacturer’s instructions. For miRNAs reverse transcription, specific primers for RNU43 (#001095, Thermo Fisher Scientific), miRNA-449a (#001030, Thermo Fisher Scientific), miRNA-449b-5p (#001608, Thermo Fisher Scientific), and miRNA-449c-5p (#241086_mat, Thermo Fisher Scientific) were used.

The complementary DNA (cDNA) was amplified by qPCR using the TaqManⓇ Universal Master Mix (Thermo Fisher Scientific) and the TaqManⓇ 20× assays for *FASN* (Hs01005622_m1, Thermo Fisher Scientific), *GAPDH* (Hs03929097_g1, Thermo Fisher Scientific), microRNA-449a (#001030, Thermo Fisher Scientific), microRNA-449b-5p (#001608, Thermo Fisher Scientific), microRNA-449c-5p (#241086_mat, Thermo Fisher Scientific, ) and RNU43 (#001095, Thermo Fisher Scientific), following the manufacturer instructions on the 9700HT Fast Real-Time PCR system (Applied Biosystems, Waltham, USA). Thereafter, the relative expression of each miRNA or mRNA was estimated through the 2^-ΔΔCt^ method, and *GAPDH* mRNA or RNU43 miRNA were used as internal controls.

### Western blot

Total protein was extracted using PierceⓇ RIPA buffer (#89900, Thermo Fisher Scientific) supplemented with protease and phosphatase inhibitor cocktail (#A32961, Thermo Fisher Scientific), according to the manufacturer’s instructions, and through sonication 10 s at 40% pulse (Sonics Vibra Cell VC 505; Sonics&Materials, Newtown, USA). Proteins were quantified using a Pierce^™^ BCA Protein Assay Kit (#23227, Thermo Fisher Scientific), according to the manufacturer’s instructions. 30 µg of total protein were separated in 6% and 8% of electrophoresis sodium dodecyl sulfate (SDS) polyacrylamide gels at a constant voltage of 120 V for 90 min. The proteins were transferred to a 0.45 μm nitrocellulose membrane (Bio-Rad, Hercules, USA) using the Trans-Blot Turbo (Bio-Rad). The membranes were then blocked with 5% bovine serum albumin (BSA) (Sigma-Aldrich, St. Louis, USA) in Tris-buffer saline-tween 20 (TBS-T; TBS 1X (Bio-Rad) and 0.1% Tween 20 (Bio-Rad)) for 1 h and incubated overnight at 4 ºC with gentle agitation with the corresponding primary antibodies: AKT (1:1000, #9272, Cell Signaling, Danvers, USA), FASN (1:1000, PA5-22061, Invitrogen), GAPDH (1:2000, MA5-15738, Invitrogen), HER2 (1:500, #2165, Cell Signaling), P44/42 MAPK (ERK1/2) (1:1000, #9102, Cell Signaling), pAKT (Tyr308) (1:1000, #9275, Cell Signaling) and pERK1/2 (Tyr 204) (1:500; sc-7383, Santa Cruz, Texas, EUA). Then, membranes were washed and incubated for 1 h at room temperature with the corresponding secondary antibodies (1:2000, anti-mouse IgG, Cell Signaling, #7076; 1:2000, anti-rabbit IgG, Cell Signaling, #7074). Pierce™ ECL Western Blotting Substrate kit (Thermo Fisher Scientific) or SuperSignal™ West Femto Maximum Sensitivity Substrate (Thermo Scientific) kit were used for developing the membranes, following the manufacturer’s instructions, in the ImageQuant™ LAS 4000 developer (GE-Healthcare Bioscience, Chicago, USA). Latterly, the chemiluminescence signal was analyzed with ImageJ-win64 software. GAPDH was used as an endogenous control.

### Luciferase reporter assay

*FASN* 3’UTR (NM_000963.2) was cloned into the pEZX-MT06 vector (Genecopoeia, Guangzhou, China). HEK293T cells were co-transfected with miRNA-449a, miRNA-449b-5p, miRNA-449c-5p, or scrambled control (100 nM), and the vector containing *FASN* 3’UTR or the pEZX-MT06 control vector (5 ng/µL). After 24 h, luciferase activity was measured using Luc-Pair^™^ Duo-Luciferase Assay Kit 2.0 (#217LF002, Genecopoeia), following the manufacturer’s instructions, and luminescence was detected in a luminescence microplate reader (LUMIstar Omega, BMG Labtech, Ortenberg, Germany). Relative luciferase activity was calculated relative to scrambled miRNA.

### In Silico analysis

The prognostic value of the miRNAs-449 (hsa-miRNA-449a, hsa-miRNA-449b-5p, hsa-miRNA-449c-5p) and *FASN* was evaluated using Kaplan-Meier plotter software (https://kmplot.com/analysis/). The miRNAs-449 and *FASN* expression was analyzed in a cohort of HER2 + BC patients (*n* = 105 and *n* = 251, respectively) with auto-selected best cut-off to evaluate its association with overall survival (OS) or disease-free survival (DFS). The Hazard ratio (HR) with 95% confidence intervals (CI) and log-rank *p-value* were calculated by the software and curves were plotted.

The GEO database (GSE24508) was used to compare the miRNAs-449 (miRNA-449a and miRNA-449b-5p) relative expression between BC (*n* = 16) tissue samples before treatment and healthy breast tissues (*n* = 22). To compare *FASN* expression between tumor samples from patients who experienced relapse (*n* = 49) compared to those who did not relapse (*n* = 34) HER2 + BC patients, the TCGA cohort was used.

DIANA-miRpath v.3 tool and TarBase v7.0. were employed to predict potential miRNAs-449 targets and to analyze biological pathways that might be modulated by them (https://dianalab.ece.uth.gr/html/mirpathv3/index.php?r=mirpath). The algorithm allows the identification of various molecular pathways controlled by different miRNAs, and statistical values are obtained through Fisher’s exact test methodology. The theoretical interaction between miRNAs-449 and the *FASN* transcript were evaluated by determining the interaction-free energies (E_int_, kJ/mol) that were calculated using the IntaRNA tool incorporated into Freiburg RNA Tools (https://rna.informatik.unifreiburg.de/IntaRNA/Input.jsp). For this, sequences of miRNAs miRNA-449a (MIMAT0001541), miRNA-449b-5p (MIMAT0003327), and miRNA-449c-5p (MIMAT0010251) (miRBase) and the 3’-UTR region of *FASN* (NM_004104.5) (NCBI) were used.

### Statistical analysis

GraphPad Prism 8.0.1 version software (La Jolla, USA) was used to perform the statistical analysis. Student’s T-test was performed to mean comparisons between the two groups. The assays were performed with technical and biological triplicates, and the data were represented as mean ± standard deviation (SD). The results were considered to be statistically significant when a *p-value* (*p*) < 0.05 was obtained: *p < 0.05, **p < 0.01, *** *p* < 0.001, and **** *p* < 0.0001.

## Results

### MiRNAs-449 are downregulated in TZ-resistant HER2 + BC cell lines and related to worse prognosis in HER2 + BC patients

**Fig. 1:**
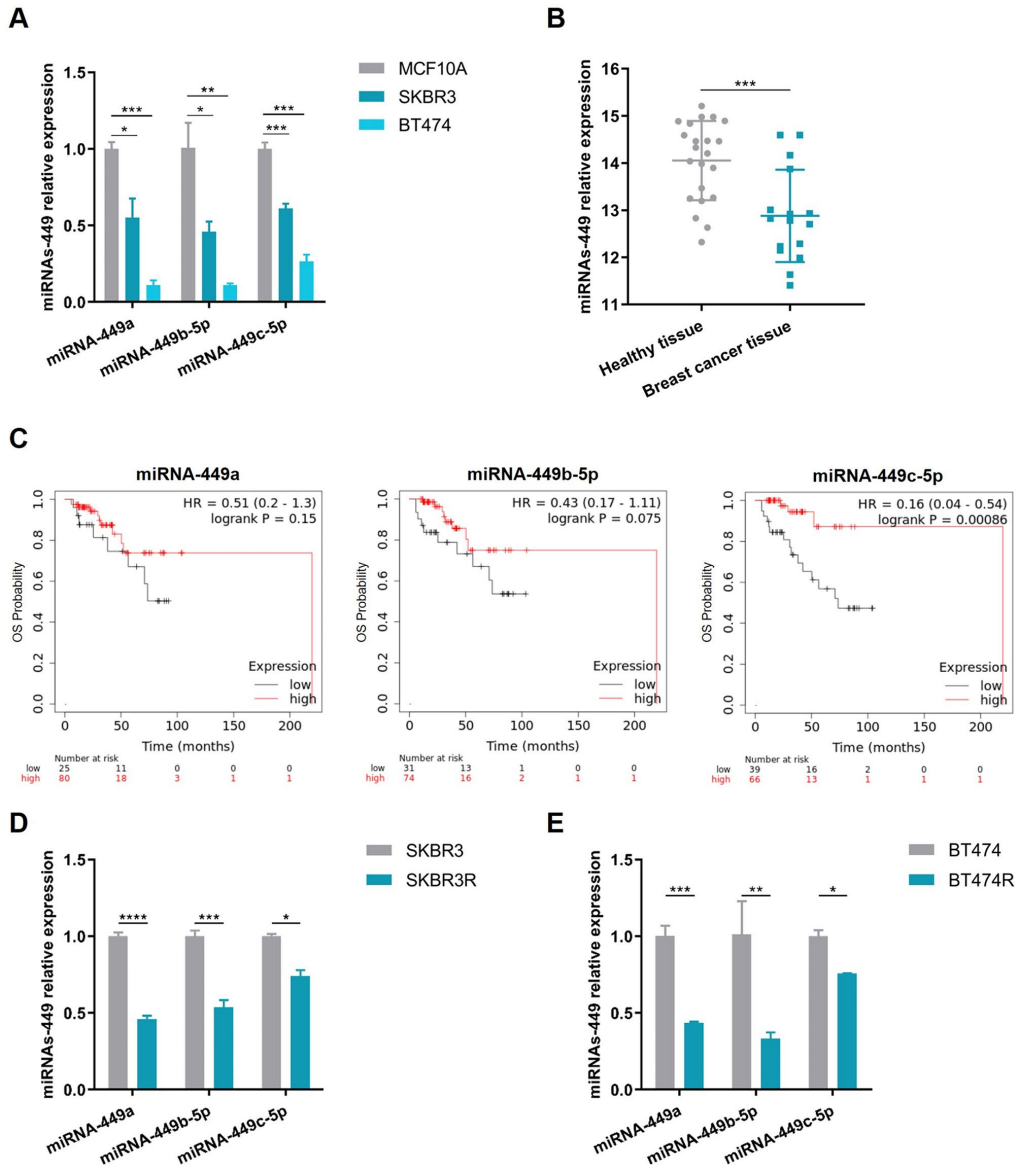
MiRNAs-449 downregulation in TZ-resistant HER2 + BC cells and related to HER2 + BC patients’ worse prognosis. **(A)** Relative expression of miRNAs-449 in MCF10A, SKBR3, and BT474 cell lines by RT-qPCR. **(B)** In silico analysis of miRNAs-449 levels between healthy tissue (*n* = 22) and BC tissue (*n* = 16) in a GEO cohort (GSE24508). **(C)** Kaplan–Meier plot of OS based on miRNAs-449 expression in a BC cohort (*n* = 105). Patients were divided into high (red) or low (black) expression by the optimal cut-off. Log-rank test *p-values* are shown. **D**,** E.** Relative expression of miRNAs-449 between SKBR3 and SKBR3R **(D)**, and between BT474 and BT474R cell lines **(E)** by RT-qPCR. * *p* < 0.05, ** *p* < 0.01, *** *p* < 0.001, **** *p* < 0.0001. HR, hazard ratio; OS: overall survival

The miRNAs-449 expression levels were determined in a non-tumorigenic breast cell line (MCF10A) and two TZ-sensitive HER2 + BC cell lines (SKBR3 and BT474). The results showed significant downregulation of miRNAs-449 in SKBR3 and BT474 compared with MCF10A (Fig. 1A). Furthermore, lower miRNAs-449 expression levels were found in BC patients’ tissue when compared with healthy tissue (*p* = 0.0006) (Fig. 1B) and it was associated with worse OS in HER2 + BC patients (miRNA-449a: *p* = 0.1500; miRNA-449b-5p: *p* = 0.0750; miRNA-449c-5p: *p* = 0.0009) (Fig. 1C**)** through in silico analyses. Furthermore, we found that miRNAs-449 were downregulated in the TZ-resistant cell lines SKBR3R and BT474R when compared to their sensitive counterparts (Fig. 1D, E). Taking these results into account, the potential role of miRNAs-449 in TZ resistance was investigated.

### MiRNAs-449 overexpression decreases cell proliferation and TZ resistance

**Fig. 2:**
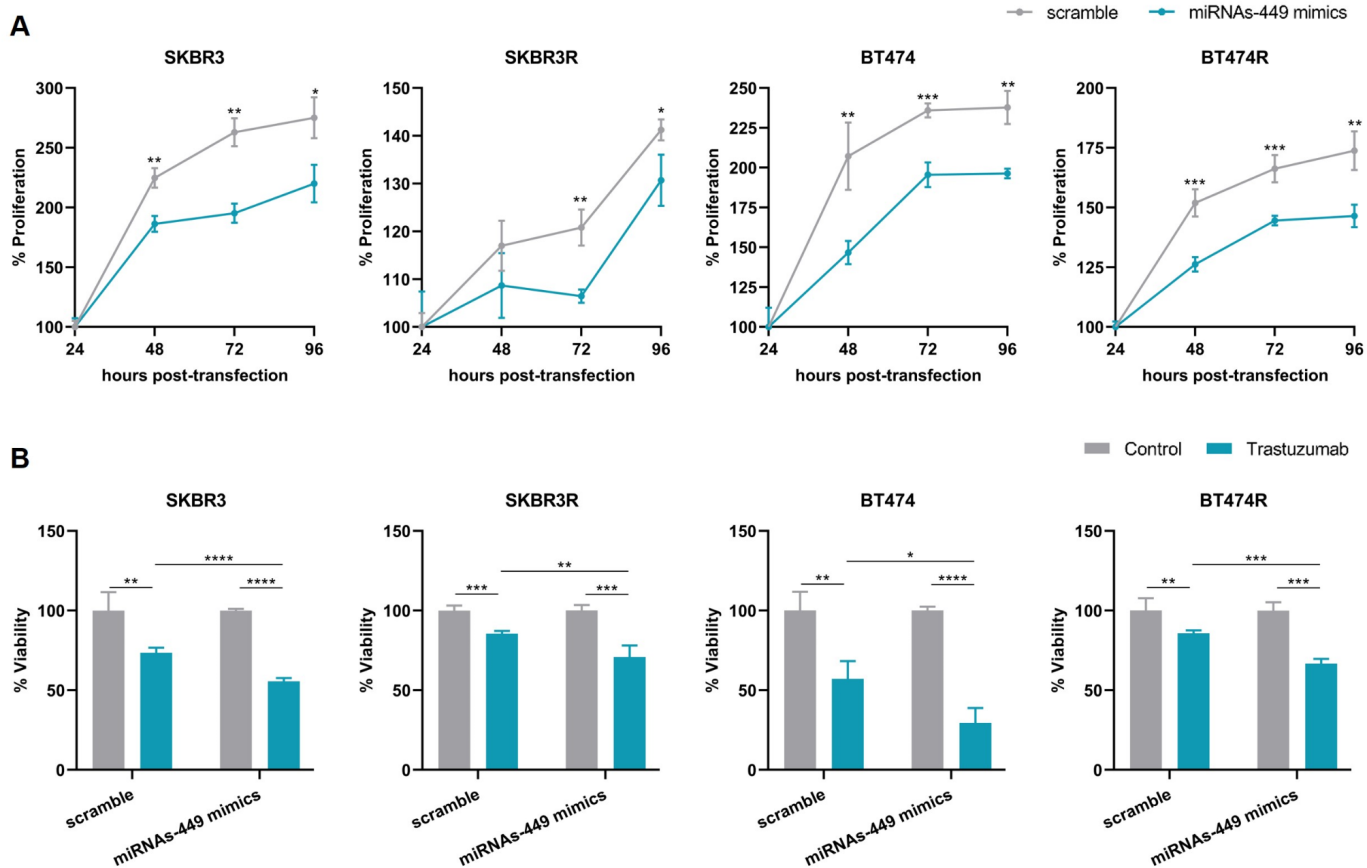
MiRNAs-449 overexpression decreases cell proliferation and TZ resistance. **A.** Cell proliferation was analyzed by WST-1 assay after 24-, 48-, 72-, and 96-hours post-transfection with miRNAs-449 mimics in SKBR3, SKBR3R, BT474, and BT474R cell lines. **B** Cell viability was analyzed by WST-1 assay after 7 days of TZ treatment and transfection with miRNAs-449 mimics in SKBR3, SKBR3R, BT474, and BT474R cell lines. * *p* < 0.05, ** *p* < 0.01, *** *p* < 0.001, **** *p* < 0.0001

Functional studies were carried out to elucidate the effect of miRNAs-449 on cell proliferation and TZ response. Our results demonstrated that the overexpression of miRNAs-449 significantly decreased cell proliferation in the sensitive and resistant HER2 + BC cell lines (Fig. 2A). Besides, overexpression of miRNAs-449 led to an increased sensitivity to TZ in both sensitive SKBR3 and BT474 cell lines, and it was able to restore the drug response in TZ-resistant cell lines (Fig. 2B).

### MiRNAs-449 regulate FASN expression in HER2 + BC

In order to predict the potential targets of the miRNA-449 family, the miRpath 2.0 software was used. This tool creates a hierarchical grouping of the miRNAs and the different biological pathways, and the resulting predicted potential pathways regulated by miRNA-449a and miRNA-449b-5p were central carbon metabolism in cancer, fatty acids biosynthesis, and fatty acid metabolism (**Supplementary Fig. 2**).

*FASN*, *Acyl-CoA Synthetase Long-Chain Family Member 4* (*ACSL4*), and *Acetyl-CoA Carboxylase Alpha* (*ACACA*) genes, which are involved in the fatty acid biosynthesis, were proposed as potential targets of the miRNA-449 family by the online tool TarBase. FASN expression was described to have a positive correlation with HER2 overexpression. Therefore, the subsequent studies were focused on miRNAs-449 modulation of TZ response through FASN.

**Fig. 3:**
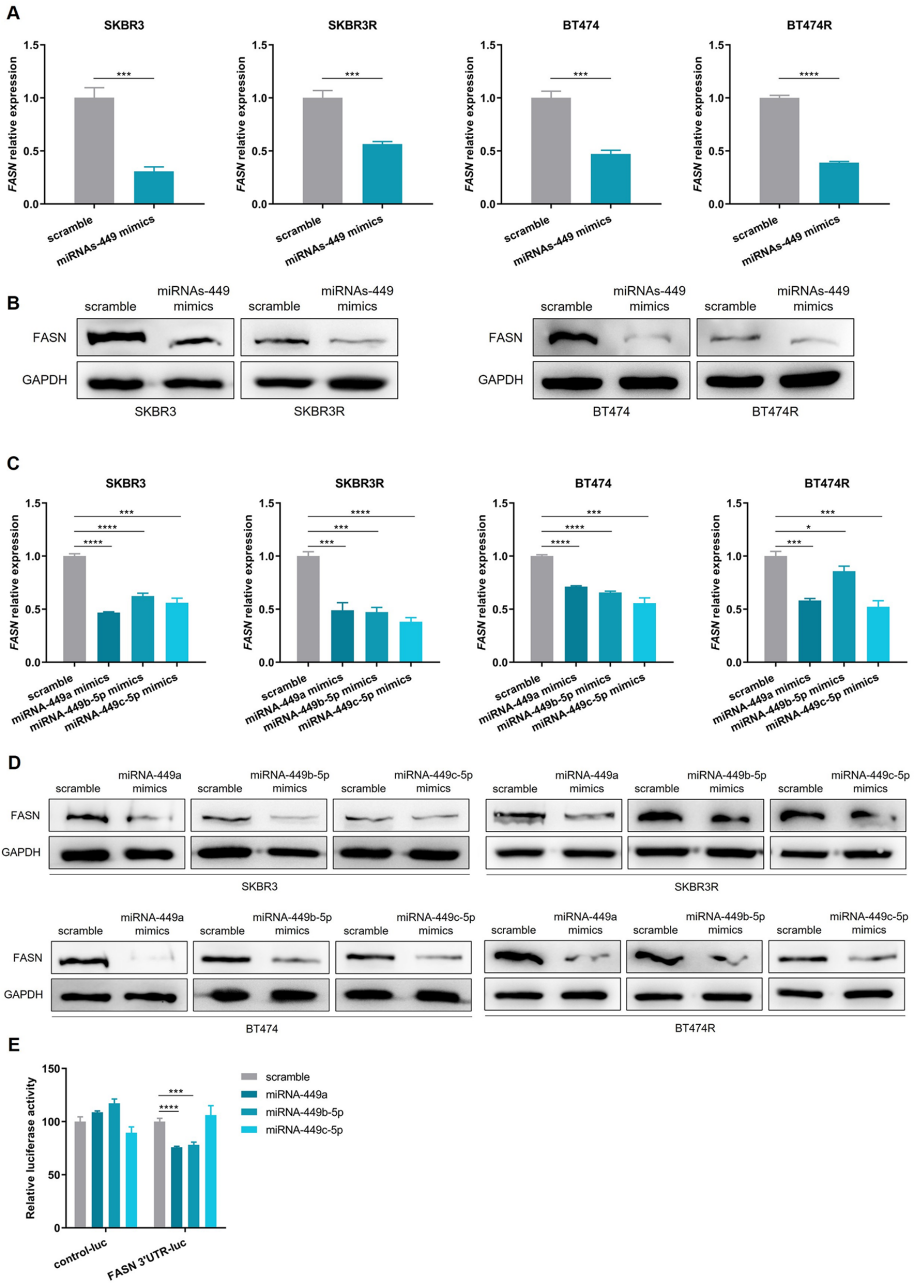
MiRNAs-449 regulate FASN expression in HER2 + BC. **A**,** B.** FASN relative expression in SKBR3, SKBR3R, BT474, and SKBR3R cells transfected with miRNAs-449 mimics by RT-qPCR (**A**) and Western blot (**B**). GAPDH was used as loading control for Western blot. **C**,** D.** FASN relative expression in SKBR3, SKBR3R, BT474, and SKBR3R cells transfected with miRNA-449a, miRNA-449b-5p, and miRNA-449c-5p mimics separately by RT-qPCR (**C**) and Western blot **D**). GAPDH was used as loading control for Western blot. **E.** Luciferase reporter assay was performed in HEK293T cell line co-transfected with luciferase vector pEZX-MT06 containing 3’UTR of *FASN* or control luciferase vector and scramble, miRNA-449a, miRNA-449b-5p, or miRNA-449c-5p mimics. * *p* < 0.05, *** *p* < 0.001, **** *p* < 0.0001

The potential targeting of *FASN* by miRNAs-449 was also confirmed by using the Freiburg RNA Tools (**Supplementary Fig. 3**). Subsequently, the effect of miRNAs-449 modulation on FASN expression was analyzed. In both sensitive and TZ-resistant cell lines, it was observed a FASN downregulation at mRNA (Fig. 3A) and protein levels (Fig. 3B) upon transfection with miRNAs-449 mimics. The same results were observed when the cells were transfected with miRNA-449a, miRNA-449b-5p, and miRNA-449c-5p mimics individually (Fig. 3C-D).

Then, to assess if *FASN* is directly modulated by miRNAs-449, the luciferase reporter assay was performed in the HEK293T cell line. The results showed a significant decrease in the luciferase activity of the cells co-transfected with *FASN* 3’UTR plasmid and miRNAs-449a (*p* < 0.0001) or miRNA-449b-5p (*p* = 0.0002) compared to scramble control. Regarding the cells co-transfected with *FASN* 3’UTR plasmid and miRNAs-449c-5p, no differences were observed. These results demonstrate that *FASN* is directly modulated by miRNA-449a and miRNA-449b-5p, but not by miRNA-449c-5p (Fig. 3E).

### FASN overexpression associates with TZ resistance and poor patients’ prognosis in HER2 + BC

**Fig. 4:**
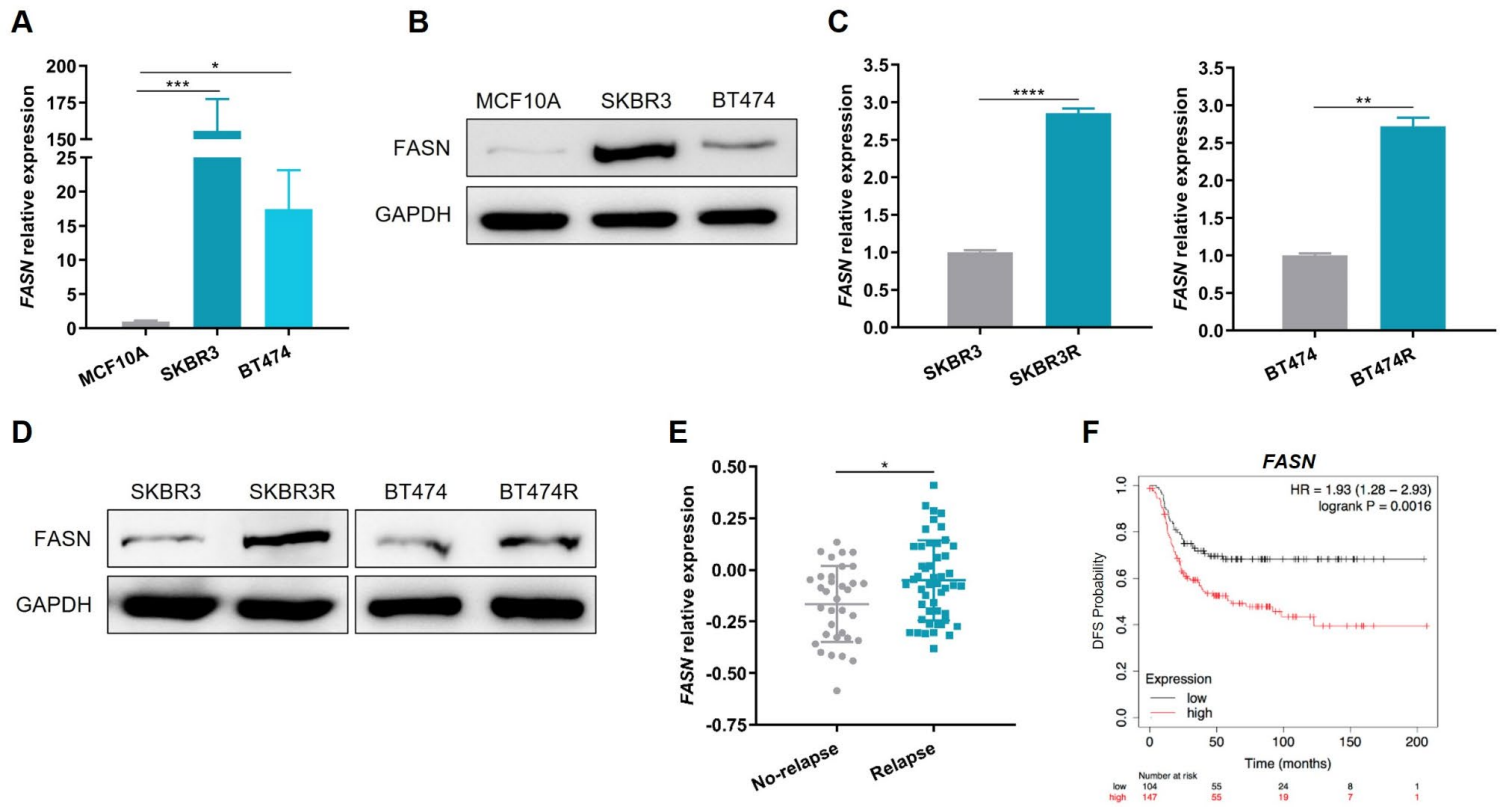
FASN overexpression associates with TZ resistance and poor patients’ prognosis in HER2 + BC. **A**,** B.** Relative expression of FASN in MCF10A, SKBR3, and BT474 cell lines by RT-qPCR (**A**) and Western blot (**B**). GAPDH was used as loading control for Western blot. **C**,** D.** FASN relative expression between SKBR3 and SKBR3R cell lines, and between BT474 and BT474R cell lines by RT-qPCR (**C**) and Western blot (**D**). GAPDH was used as loading control for Western blot. **E.** Analysis of *FASN* levels between no-relapse and relapse HER2 + BC patients from a TCGA cohort (*n* = 34 and *n* = 49, respectively). **F.** Kaplan–Meier plot of DFS based on *FASN* expression in a HER2 + BC patients TCGA cohort (*n* = 251). Patients were divided into high (red) or low (black) expression by the optimal cut-off. Log-rank test *p-values* are shown * *p* < 0.05, ** *p* < 0.01, *** *p* < 0.001, **** *p* < 0.0001. HR, hazard ratio

Firstly, FASN expression was compared between the non-tumorigenic breast cell line MCF10A and the two TZ-sensitive HER2 + BC cell lines (SKBR3 and BT474). The results showed a FASN upregulation in SKBR3 and BT474 at the mRNA level and protein levels (Fig. 4A-B). Moreover, the FASN expression was also compared between TZ-sensitive and resistant HER2 + BC cell lines. Thereby, the resistant SKBR3R and BT474R cell lines showed FASN overexpression at the mRNA and protein levels (Fig. 4C-D).

These results were also confirmed by in silico analyses of the TCGA cohort. *FASN* overexpression was found associated with relapse after TZ treatment (*p* = 0.0198) in HER2 + BC patients (Fig. 4E). Additionally, higher *FASN* levels were associated with poor DFS in HER2 + BC patients (*p* = 0.0016) (Fig. 4F), thus suggesting an inverse association between miRNAs-449 and *FASN* expression.

### *FASN* knockdown mimics the miRNAs-449 effect on cell proliferation and TZ resistance

**Fig. 5:**
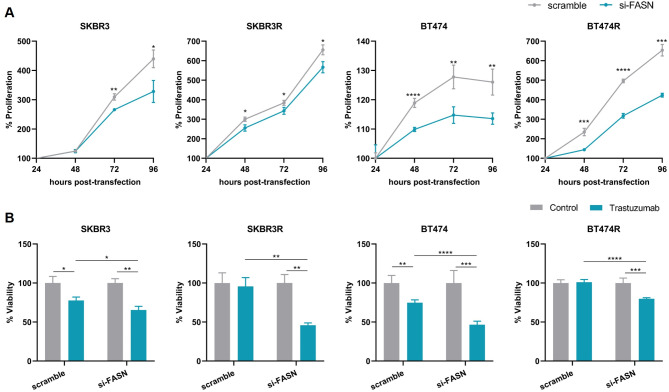
*FASN* knockdown mimics the miRNAs-449 effect on cell proliferation and TZ resistance. **(A)** Cell proliferation was analyzed by WST-1 assay after 24-, 48-, 72-, and 96-hours post-transfection with si-FASN in SKBR3, SKBR3R, BT474, and BT474R cell lines. **(B)** Cell viability was analyzed by WST-1 assay after 7 days of TZ treatment and transfection with si-FASN in SKBR3, SKBR3R, BT474, and BT474R cell lines. * *p* < 0.05, ** *p* < 0.01, *** *p* < 0.001, **** *p* < 0.0001

In order to understand whether the effect observed in cell proliferation and TZ response by miRNAs-449 is a result of FASN targeting, the HER2 + BC cell lines were transfected with si-FASN. The results showed that the downregulation of *FASN* had a significant inhibitory effect on cell proliferation in both sensitive and resistant cell lines (Fig. 5A).

Furthermore, *FASN* downregulation increased TZ response after 7 days of treatment in both sensitive and resistant cell lines (Fig. 5B). Thus, our results confirmed that FASN downregulation mimicked the effect of miRNAs-449 overexpression.

### MiRNAs-449/FASN axis regulates TZ response through PI3K/AKT pathway modulation

In order to explore the molecular pathways involved in TZ response regulation by miRNAs-449/FASN axis, alterations in protein levels of HER2 and proteins involved in the PI3K/AKT pathway were studied.

**Fig. 6:**
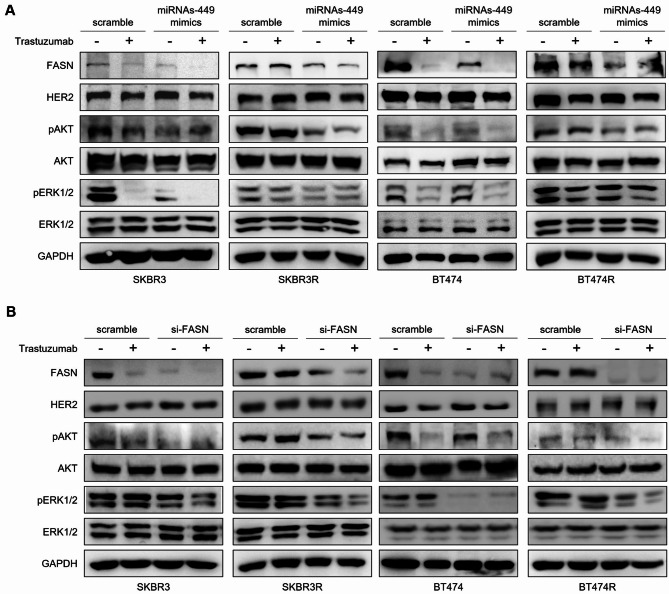
MiRNAs-449/FASN axis regulates TZ response through PI3K/AKT pathway modulation. **A**,** B.** Western blot analysis of PI3K/AKT pathway proteins, with and without 7 days of TZ treatment, in miRNAs-449 mimics transfected cells (**A**), and si-FASN transfected cells (**B**). GAPDH was used as loading control for Western blot

Firstly, it was observed that the HER2 expression was not altered by TZ treatment nor miRNAs-449 mimics in sensitive and resistant SKBR3 and BT474 cell lines. As expected, FASN expression was reduced in the cells transfected with miRNAs-449 in both sensitive and resistant cell lines. Moreover, FASN expression was downregulated upon TZ treatment only in sensitive cells, but not in SKBR3R and BT474R (Fig. 6A).

Regarding the effect in the HER2 downstream pathway PI3K/AKT, we found that levels of phosphorylated AKT and ERK1/2 were reduced by TZ addition in sensitive SKBR3 and BT474 cell lines. Nevertheless, a higher hypophosphorylation of these proteins was observed when miRNAs-449 were added in combination with TZ. By contrast, treatment with TZ alone did not affect the phosphorylation of AKT and ERK1/2 in resistant cell lines, but a hypophosphorylation was observed when resistant cells overexpressing miRNAs-449 were treated with TZ. miRNAs-449 mimics alone were able to decrease AKT and ERK1/2 phosphorylation in both sensitive and resistant SKBR3 and BT474 cell lines (Fig. 6A).

As expected, in the cells transfected with si-FASN, FASN expression was reduced in all cell lines, and the HER2 expression was consistent between conditions. Moreover, FASN expression was inhibited upon treatment with TZ, only in sensitive cell lines, Regarding PI3K/AKT HER2 downstream pathways, the results obtained in the cells transfected with si-FASN were similar to those obtained when cells were transfected with miRNAs-449 mimics (Fig. 6B).

Thus, the activation of the PI3K/AKT pathway, measured by AKT phosphorylation levels, shows no significant alterations in resistant cell lines upon TZ treatment. However, treatment with si-FASN or miRNA-449 mimics, either alone or in combination with TZ, effectively reduces AKT phosphorylation levels.

## Discussion

BC is one of the most common malignancies affecting women in the world. HER2 + BC represents approximately 15–25% of BC cases (DeSantis et al. 2015; Exman and Tolaney 2021). Currently, HER2 + BC is mainly treated with a combination of chemotherapy and anti-HER2 drugs such as TZ (Cardoso et al. 2019). Despite the use of TZ has improved the survival rate of these patients, acquired resistance to this drug remains a major challenge in the treatment of this subtype of BC. Concretely, approximately 20% of patients with HER2 + BC still develop recurrence and metastasis after adjuvant therapy (Chumsri et al. 2019; Yang et al. 2022). Nevertheless, understanding the mechanisms of the acquisition of resistance to TZ is still a challenge that must be overcome to improve BC prognosis. Therefore, this study aimed to evaluate the role of the miRNA-449 family in the modulation of HER2 + BC TZ response.

Firstly, we described that miRNAs-449 were downregulated in HER2 + BC cells compared to a non-tumorigenic cell line. Accordingly, previous research showed that miRNAs-449 are downregulated in BC (Yong-Ming et al. 2017; Jiang et al. 2019), gastric cancer (Chen et al. 2018), and liver cancer (Sandbothe et al. 2017) compared to healthy tissues. Moreover, lower levels of miRNAs-449 were associated with poorer OS in HER2 + BC patients, which suggests a relevant prognostic value of these miRNAs. In TZ-resistant HER2 + BC cell lines, we found that miRNAs-449 were downregulated when compared to sensitive cell lines. This finding suggests that miRNAs-449 might play a role in the TZ resistance development. However, as far as we know, no previous study reported any association between the miRNA-449 family and TZ resistance in HER2 + BC.

It is known that miRNAs modulate drug sensitivity and resistance through the regulation of genes involved in drug response (Garrido-Cano et al. 2022). Nevertheless, the role of miRNAs-449 in drug resistance has been studied in response to chemotherapy with cisplatin in ovarian cancer (Zhou et al. 2014). Additionally, in BC, it was reported that miRNA-449a sensitizes cells to tamoxifen (Li et al. 2018), olaparib (Vajen et al. 2022), and doxorubicin (Torres-Ruiz et al. 2024; Tormo et al. 2019). Further to this, in our study, we found that miRNAs-449 overexpression repressed cell proliferation and sensitized cells to TZ treatment. In line with this, miRNAs-449 have been shown to inhibit cell proliferation in gastric cancer (Bou Kheir et al. 2011) and BC (Yong-Ming et al. 2017). This suggests that miRNAs-449 might be a potential therapeutic target for improving TZ response in HER2 + BC patients.

To our knowledge, this study was the first to identify and confirm that miRNAs-449 target *FASN*, thus highlighting the relevance of fatty acid metabolism-related pathways in HER2 + BC. FASN is the most extensively studied lipogenic enzyme in the context of carcinogenesis. Cancer cells show increased lipogenic activity and cholesterol levels due to their greater need for membrane components, energy storage, and activation of lipid raft-dependent signaling pathways (Monaco 2017). FASN catalyzes the conversion of malonyl-coenzyme A (CoA) and acetyl-CoA into palmitic acid, a 16-carbon chain saturated fatty acid that acts as a precursor of fatty acids, thus giving tumor cells a proliferative advantage (Ventura et al. 2015). In addition, FASN has been described as a new mechanism of multidrug resistance that involves changes in plasma membrane properties that protect cells from endogenous and exogenous factors (Rysman et al. 2010). Furthermore, FASN modifies the BC cells for drug-induced apoptosis due to an overproduction of palmitic acid (Liu et al. 2008). As a result of an increase in lipogenesis and the changes in cell death susceptibility, FASN might limit the uptake of chemotherapeutics such as doxorubicin and make cancer cells less susceptible to cytotoxic agents (Rysman et al. 2010).

Our results demonstrated for the first time that miRNA-449a and miRNA-449b-5p directly target the 3’UTR of *FASN*. These could be explained because miRNA-449a and miRNA-449b-5p share similar binding specificity between them, whereas miRNA-449c-5p might regulate *FASN* expression indirectly.

Recent studies have demonstrated a strong correlation between FASN overexpression and HER2 amplification in BC. Particularly, in a cohort of 189 patients with invasive breast carcinoma, 85% of HER2 + tumors were scored with high *FASN* expression (Corominas-Faja et al. 2017). Moreover, the HER2-FASN correlation was observed in HER2 + gastric cancer, in which *FASN* upregulation was correlated with cancer cell stemness and poor prognosis (Castagnoli et al. 2023). In the context of HER2 + BC, *FASN* upregulation was also related to cancer progression (Jin et al. 2010). Besides, in silico analysis showed an association between *FASN* overexpression and relapse in a HER2 + BC patients’ cohort after TZ treatment. Furthermore, our study revealed a higher *FASN* expression in TZ-resistant HER2 + BC cell lines compared with sensitive cells. These results indicate that FASN might be involved in TZ response regulation by miRNAs-449 in HER2 + BC.

Our hypothesis was confirmed as FASN loss-of-function experiments mimicked the effect of miRNAs-449 overexpression on cell proliferation and TZ resistance, thus suggesting that miRNA-449 might regulate TZ response through FASN-mediated pathways in HER2 + BC. These results are consistent with previous works in BC where treatment with FASN inhibitors (EGCG and G28UCM) in combination with pertuzumab was able to resensitize lapatinib plus TZ-resistant cells to anti-HER2 drugs (Blancafort et al. 2015). Remarkably, our study demonstrated that FASN inhibitors restored sensitivity to TZ in HER2 + BC cells with acquired TZ resistance, which is also consistent with previous research regarding the restoration of sensitivity of HER2 + BC to TZ and lapatinib after FASN inhibition (Vazquez-Martin et al. 2007). As well, in HER2 + gastric cancer models, the combination of TZ with the FASN inhibitor TVB3166 reverted the anti-HER2 therapy resistance (Castagnoli et al. 2023).

Finally, we investigated the potential mechanisms underlying the regulation of TZ response by the miRNA-449 family. We proved that the miRNAs-449/FASN axis regulates TZ response through the PI3K/AKT pathway. This pathway is the most relevant in HER2-mediated signaling in BC, unlike other pathways, for several reasons. Overexpression or amplification of HER2 activates PI3K, which phosphorylates PIP2 to PIP3, recruiting and activating AKT. This pathway is crucial for cell survival, proliferation, and growth, as AKT phosphorylates targets that inhibit apoptosis and promote cell cycle progression. In HER2 + BC, PI3K/AKT signaling is often hyperactivated, driving tumorigenesis and therapy resistance. Common genetic alterations, such as PIK3CA mutations or PTEN loss, further amplify this pathway. Furthermore, PI3K/AKT activation contributes to resistance against HER2-targeted therapies by circumventing HER2 blockade. This is consistent with previous studies that have shown that the PI3K/AKT pathway is involved in the regulation of TZ response in HER2 + BC due to HER2/HER3 heterodimer formation which leads to the activation of this pathway (Junttila et al. 2009).

Some studies indicate that the modulation of the PI3K/AKT pathway is mediated by a crosstalk between HER2 and FASN. Indeed, transcriptome analysis demonstrated an increase of FASN in HER2 + BC cell lines in comparison with HER2-negative cells, and a reduction of FASN expression as a consequence of HER2 inhibition (Kumar-Sinha et al. 2003). Taking this into account, it was hypothesized that FASN modulation may have an impact on HER2 expression with consequent modulation of downstream pathways. A previous study in BC cells showed that *FASN* inhibition suppresses HER2 overexpression by attenuating the promoter activity of the *HER2* gene, which leads to a reduction of its transcription rate (Vazquez-Martin et al. 2007). However, our results indicate that the regulation of PI3K/AKT pathway by the miRNAs-449/FASN axis occurs in a HER2-independent manner once no alteration in HER2 protein levels was observed as a consequence of miRNAs-449 and *FASN* modulation. Therefore, another hypothesis needs to be considered. Our study demonstrated that downregulation of *FASN* through either miRNAs-449 mimics or *FASN* silencers leads to the inactivation of PI3K/AKT HER2 downstream pathways by reducing phosphorylation of AKT and ERK, even in acquired TZ-resistant cells. These results are consistent with previous studies that reported that FASN alters HER2 downstream pathways such as PI3K/AKT (Blancafort et al. 2015).

Altogether, our data suggest that the miRNA-449 family inhibits the PI3K/AKT signaling pathway through FASN targeting, improving the TZ treatment efficacy of HER2 + BC. Beyond the data presented in this study, further in vitro and in vivo validation are necessary to confirm the role of FASN in TZ resistance. This validation is crucial to translate these findings into future clinical studies and to explore the potential of targeting FASN as a therapeutic strategy to overcome TZ resistance in HER2 + BC patients.

## Conclusion

In conclusion, our study provides evidence for the miRNAs-449 involvement in TZ resistance in HER2 + BC. The identification of the miRNA-449 family as a potential therapeutic target for improving TZ response in HER2 + BC patients is supported by previous studies on the role of these miRNAs in response to other cancer treatments and drug sensitization. Our findings also suggest that the TZ response regulation by miRNAs-449 might involve FASN-mediated pathways, namely the PI3K/AKT pathway. These insights into the molecular mechanisms of TZ resistance might aid in developing more effective therapeutic strategies for HER2 + BC. By providing a rationale for FASN inhibitors in combination with anti-HER2 agents, our research offers a potential avenue for overcoming TZ resistance and improving treatment options for HER2 + BC patients.

## Electronic supplementary material

Below is the link to the electronic supplementary material.

Supplementary Material 1: Supplementary fig. 1 Confirmation of transfection with mimics of miRNAs-449 and si-FASN. Supplementary Fig. 2 Predicted biological pathways regulated by miRNAs-449. Supplementary Fig. 3 Interaction between miRNAs-449 and 3’-UTR region of *FASN* mRNA.

## Data Availability

No datasets were generated or analysed during the current study.

## Abbreviations

ACACA: Acetyl–CoA Carboxylase Alpha
ACSL4: Acyl–CoA Synthetase Long–Chain Family Member 4
ADAM22: ADAM Metallopeptidase Domain 22
AKT: Protein kinase B
BC: Breast cancer
BRCA2: Breast Cancer Gene 2
BSA: Bovine serum albumin
CDC20B: Cell division cycle 20B
CI: Confidence intervals
CoA: Coenzyme A
DFS: Disease–free survival
DMEM: F12–Dulbecco’s Modified Eagle Medium F12
E2F3: E2F Transcription Factor 3
FASN: Fatty Acid Synthase
FBS: Fetal Bovine Serum
HER2: Epidermal Growth Factor Receptor 2
HER2+: HER2–positive
HR: Hazard ratio
MAPK: Mitogen–activated Protein Kinase
miRNA: MicroRNA
mRNA: Messenger RNA
OS: Overall survival
PI3K: Phosphatidyl Inositol 3 Kinase
SD: Standard deviation
SDS: Sodium dodecyl sulfate
TBS: T–Tris–buffer saline–tween 20
TZ: Trastuzumab
