# Supplementary material for: Involvement of microRNAs-449/FASN axis in response to trastuzumab therapy in HER2-positive breast cancer
Source: Mol Med. 2025 Mar 25;31:116. doi: 10.1186/s10020-025-01163-z (PMC11938741; doi:10.1186/s10020-025-01163-z)
Supplement: Supplementary file 1 — Supplementary Material 1: Supplementary fig. 1 Confirmation of transfection with mimics of miRNAs-449 and si-FASN. Supplementary Fig. 2 Predicted biological pathways regulated by miRNAs-449. Supplementary Fig. 3 Interaction between miRNAs-449 and 3’-UTR region of FASN mRNA. [file 10020_2025_1163_MOESM1_ESM.docx]

**Involvement of microRNAs-449/FASN axis in response to trastuzumab therapy in HER2-positive breast cancer**

Ana Lameirinhas^1+^, Sandra Torres-Ruiz^1+^, Iris Garrido-Cano^1,2^, Cristina Hernando^1,3^, María Teresa Martínez^1,3^, Ana Rovira^4,5,6^, Joan Albanell^4,5,6^, Sandra Zazo^4,7^, Federico Rojo^4,7^, Begoña Bermejo^1,3,4^, Ana Lluch^1,3,4,8^, Juan Miguel Cejalvo^1,3,4^, Eduardo Tormo^1,4*^, Pilar Eroles^1,4,9*^

1. INCLIVA Biomedical Research Institute, Valencia 46010, Spain.

2. Instituto Interuniversitario de Investigación de Reconocimiento Molecular y Desarrollo Tecnológico (IDM), Universidad Politécnica de València, Universidad de Valencia, Valencia 46022, Spain.

3. Department of Medical Oncology, Hospital Clínico Universitario de València, Valencia 46010, Spain.

4. Center for Biomedical Network Research on Cancer (CIBERONC), Madrid 28019, Spain.

5. Department of Medical Oncology, Hospital del Mar, Barcelona 08003, Spain.

6. Cancer Research Program, IMIM (Hospital del Mar Medical Research Institute), Barcelona 08003, Spain.

7. Department of Pathology, Fundación Jiménez Díaz, Madrid 28040, Spain.

8. Department of Medicine, Universidad de Valencia, Valencia 46010, Spain.

9. Department of Physiology. Universidad de Valencia, Valencia 46010, Spain.

+ Equal contribution

*Corresponding authors:

PE: pilar.eroles@uv.es; ET: tormared@gmail.com


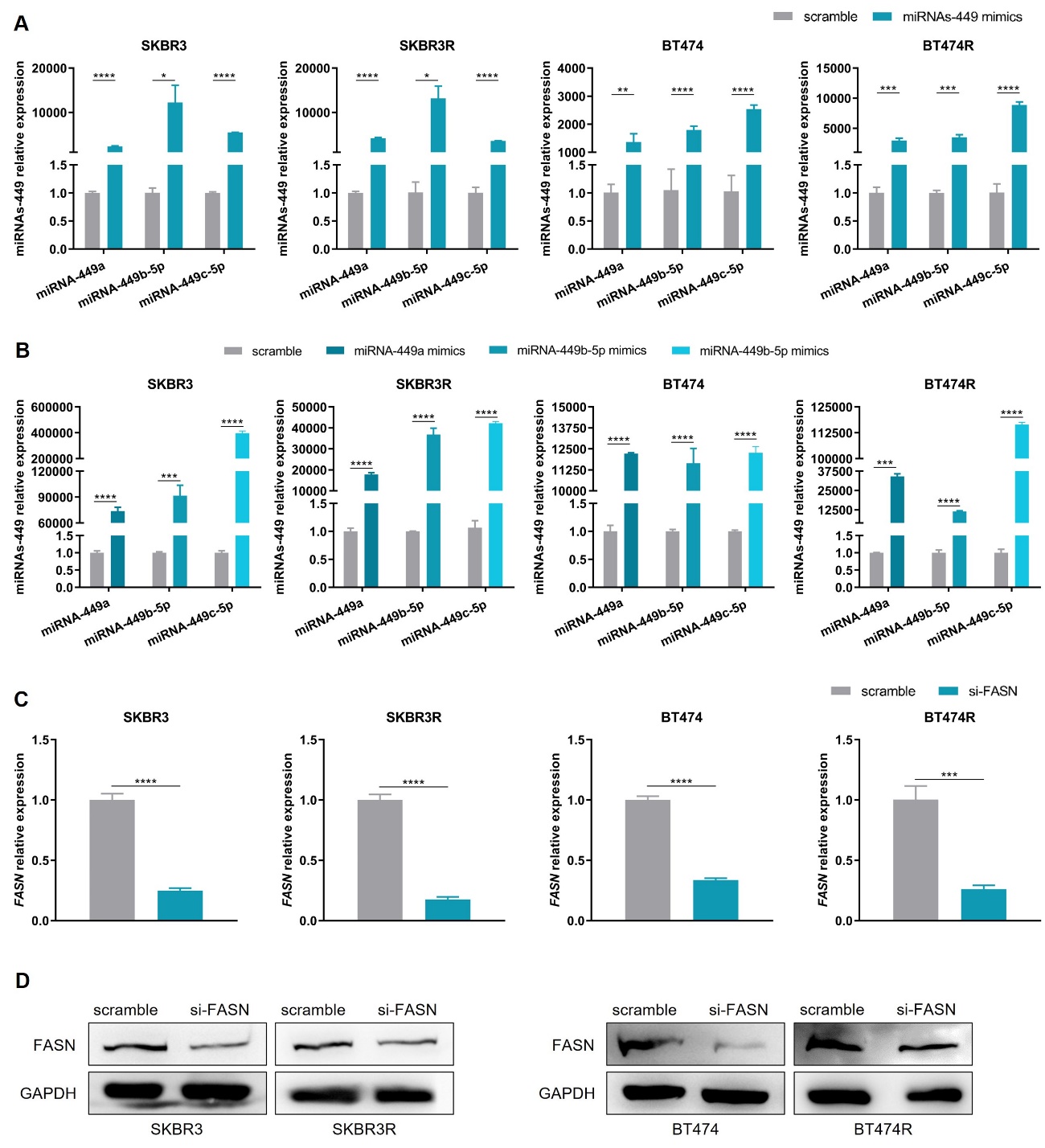


**Supplementary Fig. 1. Confirmation of transfection with mimics of miRNAs-449 and si-FASN. A.** Relative expression of miRNAs-449 by RT-qPCR in SKBR3, SKBR3R, BT474, and BT474R cells transfected with miRNAs-449 mimics compared to the control. **B.** Relative expression of miRNAs-449 by RT-qPCR in SKBR3, SKBR3R, BT474, and BT474R cells transfected with miRNA-449a, miRNA-449b-5p, and miRNA-449c-5p mimics separately compared to the control. **C, D.** FASN relative expression in SKBR3, SKBR3R, BT474, and BT474R cells transfected with si-FASN compared to the control by RT-qPCR (C) and Western blot (D). GAPDH was used as loading control for Western blot. * *p* < 0.05, ** *p* < 0.01, *** *p* < 0.001, **** *p* < 0.0001.

**
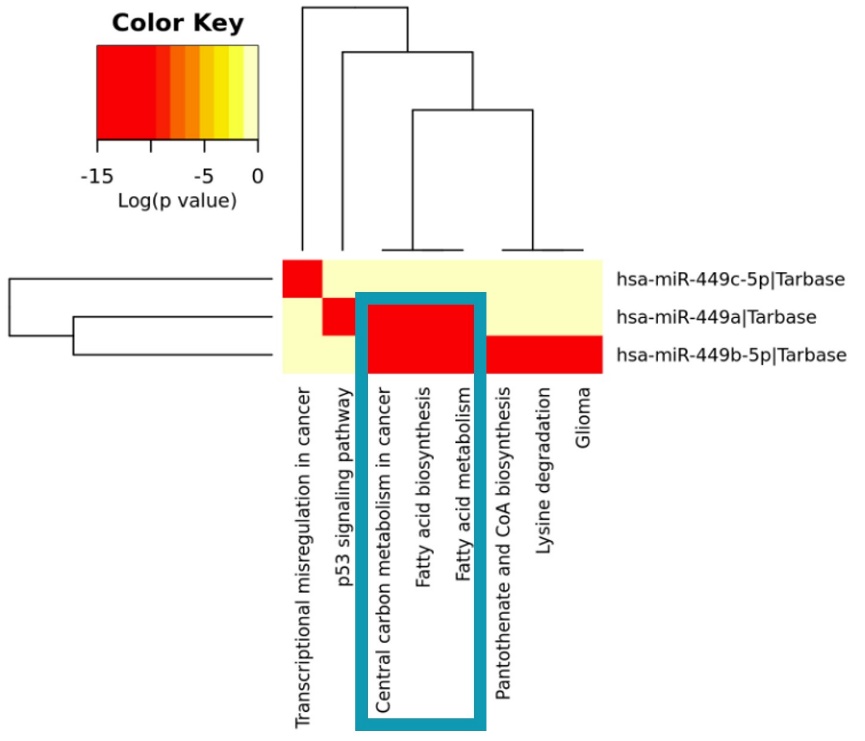
**

**Supplementary Fig. 2. Predicted biological pathways regulated by miRNAs-449*.***  Biological pathways enriched in the genes predicted as targets of miRNAs-449 by miRpath 2.0 software. The red legend indicated in the figure corresponds to the *p-values* calculated through Fisher's exact test.

**
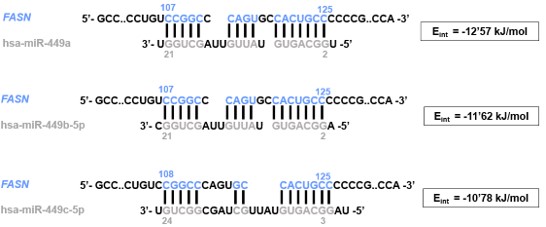
**

**Supplementary Fig. 3. Interaction between miRNAs-449 and 3’-UTR region of *FASN* mRNA***.* Theoretical binding regions between *FASN* 3’UTR and each of the studied miRNAs (hsa-miRNA-449a, hsa-miRNA-449b-5p, and hsa-miRNA-449c-5p). The free energy (Eint, kJ/mol) calculated for each of the interactions is indicated. Source: IntaRNA Freiburg RNA Tools.
